# Effect of Injection Molding Conditions on Crystalline Structure and Electrical Resistivity of PP/MWCNT Nanocomposites

**DOI:** 10.3390/polym12081685

**Published:** 2020-07-28

**Authors:** Marta Zaccone, Ilaria Armentano, Federico Cesano, Domenica Scarano, Alberto Frache, Luigi Torre, Marco Monti

**Affiliations:** 1 Proplast, Via Roberto di Ferro 86, 15122 Alessandria (AL), Italy; marta.zaccone@proplast.it; 2 Department of Applied Science and Technology, Polytechnic of Turin, Corso Duca degli Abruzzi 24, 10129 Torino, Italy; alberto.frache@polito.it; 3 Department of Economics, Engineering, Society and Business Organization (DEIM), Tuscia University, 01100 Viterbo, Italy; 4 Department of Chemistry, University of Turin, Via P. Giuria, 7, 10125 Torino, Italy; federico.cesano@unito.it (F.C.); domenica.scarano@unito.it (D.S.); 5 Department of Civil and Environmental Engineering, University of Perugia, Strada di Pentima 4, 05100 Terni, Italy; luigi.torre@unipg.it

**Keywords:** polymer nanocomposites, carbon nanotubes, electrical properties, crystallinity, injection molding, processing

## Abstract

Polypropylene (PP) / multi-walled carbon nanotube (MWCNT) nanocomposites were prepared by melt-mixing and used to manufacture samples by injection molding. The effect of processing conditions on the crystallinity and electrical resistivity was studied. Accordingly, samples were produced varying the mold temperature and injection rate, and the DC electrical resistivity was measured. The morphology of MWCNT clusters was studied by optical and electron microscopy, while X-ray diffraction was used to study the role of the crystalline structure of PP. As a result, an anisotropic electrical behavior induced by the process was observed, which is further influenced by the injection molding processing condition. It was demonstrated that a reduction of electrical resistivity can be obtained by increasing mold temperature and injection rate, which was associated to the formation of the γ-phase and the related inter-cluster morphology of the MWCNT conductive network.

## 1. Introduction

In the last decades, an increasing interest in electrically conductive polymer composites has been observed, with special focus on traditional insulating polymers filled with electrically conductive additives [1]. Their enhanced electrical conductivity allows them to compete with metals, taking advantage of the peculiar properties of polymers, such as low cost, easy processability, lightweight, and resistance to chemical corrosion. 

Among electrically conductive additives, carbon-based nanomaterials have attracted considerable attention [2]. In particular, carbon nanotubes (CNTs) have been identified as a very promising filler to obtain high-performance nanocomposites [3,4]. Due to their chain-like aggregated structure, they tend to easily form a conductive path within the polymer matrix, which is more effective if compared with other conductive additives, and which is well-described by the percolation theory [5,6,7]. For this reason, they can be exploited for a variety of industrial applications, such as, sensors and electromagnetic interference (EMI) shielding devices [8,9,10,11]. Moreover, CNTs have reached a technological maturity and a cost-to-performance ratio, which is suitable for a real market exploitation, in particular, in the case of multi-walled carbon nanotubes (MWCNTs) [12].

Many factors are known to affect the electrical resistivity of CNT-polymer nanocomposites, such as the content of CNTs and their properties (e.g., type of CNTs, aspect ratio, morphology and surface functionalization degree), the type of polymer matrix, together with mixing and shaping process [3,7,13]. Many studies have been focused on the relationship between processing conditions and electrical properties, in order to obtain highly efficient CNT nanocomposites in terms of low electrical resistivity and percolation threshold [14,15,16,17]. As for the mixing process, it has been widely reported that MWCNT dispersion is a key point in determining the electrical performance of the resulting nanocomposites [15,16]. Moreover, several studies report the relevance of the shaping phase, which typically occurs when the polymer is in the molten state in the resulting morphology [17,18]. 

Injection molding is generally considered one of the most used techniques for manufacturing plastic components. During the processing phase, the longitudinal flux of the molten polymer can induce an orientation in fiber-like additives, both micron-sized, like carbon and glass fibers, and in nano-sized particles, like CNTs [19]. Moreover, due to the effect of the shear stress gradient in the through-thickness direction, which is present during the shaping phase, a skin-core morphology of injection-molded polymer nanocomposites is typically obtained [20,21].

Since the morphology of an injection-molded MWCNT-polymer nanocomposite is strictly connected to the process itself, several studies have been published regarding how the processing conditions can affect the morphology and the related electrical properties [18,22,23,24,25,26]. Several papers deal with amorphous matrices. As an example, Villmow et al. [23] studied the correlation between process parameters and the resulting electrical resistivity of polycarbonate-based CNT nanocomposites by varying holding pressure, injection velocity, mold and melt temperature. Other studies have focused their attention on semi-crystalline matrices [25,26]. As an example, Ameli et al. [25] developed injection-molded PP/CNT nanocomposite foams and found that conductivity is favored by an increase in the injection flow rate, and also influenced by foaming parameters. 

Although several studies focused on the relationship between processing condition and crystalline structure [27,28,29,30], to the best of our knowledge, only few attempts have been carried out to correlate the specific crystalline morphology of nanocomposites based on electrically conductive nanofillers and the resulting electrical properties [31,32,33,34,35]. As an example, Von Baeckmann et al. [27] recently studied the correlation between processing condition and resulting crystalline structure, finding that the PP γ-phase is favored as the cooling rate is reduced. As for the correlation with electrical properties of nanocomposites, it is worth mentioning the study of Kazemi et al. [31], in which they have been able to tune the matrix’s crystal type of PP/CNT composites by isothermal annealing under supercritical CO_2_ conditions, and verifying the effect on the electrical properties. Moreover, Kalaitzidou et al. [32] studied graphene nano platelets xGnP^®^/PP nanocomposites, discovering that the presence of xGnP^®^ significantly alters the crystallization behavior of PP and the mechanical and electrical properties can be strongly modified by tuning the processing condition. Finally, Wang et al. [35] studied the crystal-nucleating capability of CNTs in PP as controlled through the addition of a sorbitol-based external nucleating agent and via controlling the cooling rate, highlighting the positive effect in the conductivity using slow cooling in addition to annealing treatment. However, to the best of our knowledge, all the results reported in literature, which connect the cooling rate to the crystalline structure and the electrical properties, barely take into account processing conditions and the industrial feasibility of the technical approach as whole. 

In this paper, we perform a study on PP/MWCNT nanocomposites, focused on the correlation of the injection-molding processing conditions (i.e., mold temperature and injection flow rate) with the resulting specific crystalline structure and the electrical behavior.

## 2. Materials and Methods 

### 2.1. Materials

Polypropylene (PP) Moplen RP348R (Lyondell Basell) was selected as a polymer matrix. It is a random copolymer (injection molding grade), with a MFI of 25 g/10 min (230 °C–2.16 kg) and a density of 900 kg/m^3^. Multi-walled carbon nanotubes, Nanocyl NC7000, produced by Nanocyl via catalytic carbon vapor deposition (CCVD) process, were used as conductive nanofillers. As reported by the manufacturer, they are about 10 nm in diameter and 1.5 µm in length. Their surface area is 250–300 m^2^/g. The percentage of pure carbon in their composition is 90%.

### 2.2. Processing

Several MWCNT contents (namely: 1–2–3–4–5–6–7 wt%) were homogeneously mixed with PP by using a co-rotating twin-screw extruder, Leistritz 27E. The screws have a diameter (D) of 27 mm and a length-to-diameter ratio (L/D) equal to 40. The screw speed was maintained constant at 220 rpm. The temperature profile was set in the range of 190–200 °C. 

**Figure 1:**
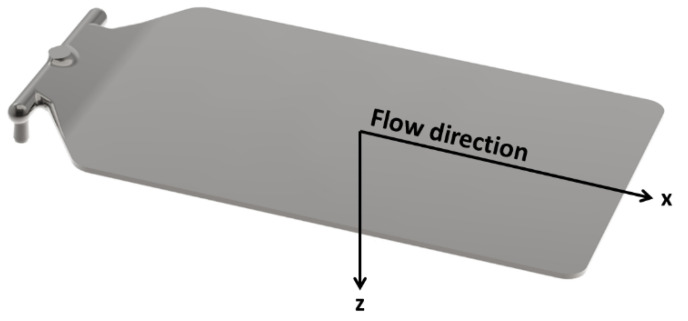
Schematic sketch of the sample with the testing and flow directions.

The obtained nanocomposites were injection molded, using a Ferromatik K - Tec 200 press, with a screw diameter D of 50 mm. Rectangular-shaped samples (100 × 140 × 2 mm^3^) were prepared. Figure 1 reports a sketch of the sample, with the directions used to perform the electrical characterization and the flow during the injection molding process. As a standard processing condition, the temperature of the mold was set at 25 °C and the flow rate of the molten polymer inside the cylinder toward the mold (i.e., the injection rate) at 70 cm^3^/s. These parameters are referred to as standard condition hereinafter. 

**Figure 2:**
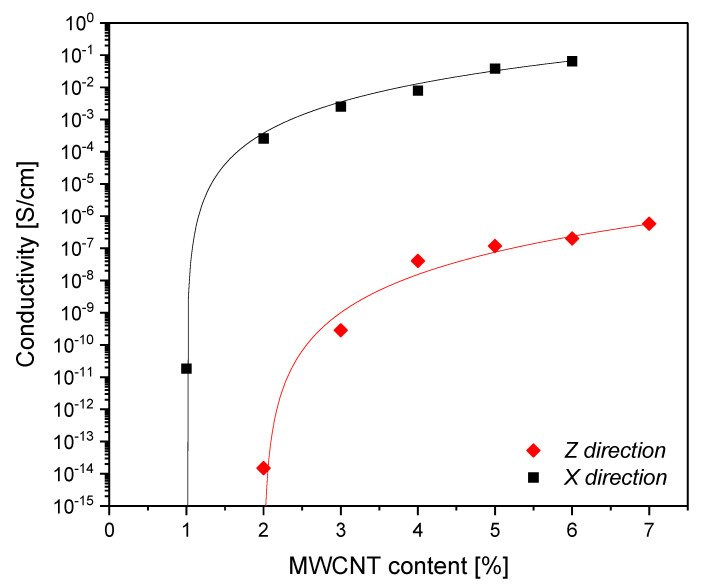
Percolation curves in X and Z directions: experimental data and fitting curves.

Starting from the standard condition, a series of specific injection-molding tests was performed, changing both the injection rate and the mold temperature. In particular, the temperature of the mold was also set at 70 °C and 100 °C, and the injection rate at 35 cm^3^/s and 250 cm^3^/s. The mold temperature equal to 100 °C is considerably higher if compared to the typical settings for PP and it has been obtained through the so-called Heat and Cool (H and C) process. This process was obtained by using a Vario-Therm control unit (HB-Therm), kindly supplied by Nickerson Italia. It consists in a dynamic control of the temperature of the mold, which is heated to a high temperature value during the injection phase (allowing the lowering of the viscosity that facilitates the process) and quickly cooled during the packing phase (avoiding the possible degradation of the molten material and reducing the cycle time–thus improving the competitiveness of the process). Processing parameters were changed for the MWCNT-based formulations in the range of the percolation threshold (2–4%), as obtained in a previous study from the same authors [21] and confirmed by the results that will be shown in Figure 2. 

### 2.3. Electrical Properties

The evaluation of the electrical properties was carried out by performing both bulk and surface DC resistivity tests, according to ASTM D257 standard. Bulk measurements were performed in the two main directions (X and Z), as reported in the scheme in Figure 1: longitudinal to the flux of the material during the mold filling (X), hereinafter referred to as in-plane direction, and in the through-thickness direction (Z). The bulk tests in the Z direction and the surface resistivity tests were performed using a Keithley 6517B electrometer, with the Keithley 8009 test fixture. 

The electrical properties in the X direction were measured using a Keithley 2420 instrument. Each measurement was repeated three times with a standard deviation less 5% for each sample.

### 2.4. Morphology

The morphology and the structure of the resulting nanocomposites have been studied according to two different approaches. On the one hand, optical and field emission scanning electron (FESEM) microscopy has been used to investigate the morphology of the MWCNT clusters. On the other hand, X-ray diffraction (XRD) techniques have been used to highlight the role of the crystalline structure of PP on the morphology and electrical properties of the produced samples.

The optical microscopy was performed through an Axioskope 2 Zeiss microscope by means of polarized light on cryo-microtomed slices, which have been previously obtained using an Ultracut E Reichert Jung ultracryomicrotome instrument and a Camera FC4, in order to achieve the correct cutting temperature. FESEM images were obtained by using a Zeiss Merlin 4248 instrument. The specimens were preventively cryo-fractured in liquid nitrogen and then coated with a thin layer (<10 nm) of Chromium before observation. 

X-ray diffraction (XRD) analysis was performed using a Panalytical X’Pert PRO (Cu Kα radiation, wavelength of 1.54187 Å) diffractometer, with 2θ ranging from 2° to 30° (step rate of 0.026°/min). Each sample was tested three times. As for the calculation of the crystalline fractions, the experimental error due to the baseline evaluation is typically estimated in the range of 5–10% of the calculated value.

### 2.5. Rheology

Rheological characterization was performed in nitrogen atmosphere on a strain-controlled rotational rheometer (Rheometric Scientific ARES, model 2KFRT), using the parallel-plate geometry. Test specimens were compression molded in disks (1 mm thickness) of 25 mm diameter. Frequency sweep tests were performed in the 0.03–100 rad/s frequency range, with a fixed strain of 5% (as determined by a preliminary strain sweep test–data not shown). The test temperature was set at 200 °C. Each measurement was repeated three times.

### 2.6. Differential Scanning Calorimetry

Differential scanning calorimetry (DSC) was performed on a representative specimen cut from the whole cross-sectional area of the injection-molded sample. A Q800 equipment, from TA Instruments, was used. A single heating scan was set from 25 to 190 °C, with a heating rate of 10 °C/min. The degree of crystallinity was calculated according to the equation,

(1) $$X_{c}=\frac{100}{100-\mathrm{wt}\%}\frac{\Delta H_{m}}{\Delta H_{0}}$$

where *X_c_* is the crystalline fraction of the matrix, wt% is the MWCNT content, and *ΔH*_0_ is the theoretical crystallization enthalpy of 100% crystalline PP. Two different *ΔH*_0_ have been taken into account, for the two different PP crystal phases, namely 209 J/g for α-phase and 190 J/g for γ-phase [27]. Each measurement was repeated three times and the reported value of crystallinity is an average of the performed measurements.

## 3. Results and Discussion

### 3.1. Electrical Properties

Close to the electrical percolation threshold, electrical conductivity shows by definition a sudden rise of several orders of magnitude [21,36,37]. In this study, the electrical percolation curves obtained with the defined standard condition in the in-plane (X) and through-thickness (Z) directions (Figure 2) were fitted by the well-known power law,

(2) $$\sigma =\sigma_{0}{\left(\varphi -\varphi_{c}\right)}^{t}$$

where *σ*_0_ is a parameter related to the electrical conductivity of the filler, *φ*_c_ is the percolation threshold and *t* is the conductivity exponent [38]. The percolation threshold occurs at lower MWCNT content in the X direction with respect to the Z direction. The conductivity exponent *t* is related to the network structure of the percolative path [39]. A high conductivity exponent (values over 2) indicates that the conductive nanofillers form a more uniform and connected path after percolation [40]: observing the calculated *t* values in the X and Z directions (3.2, and 3.9, respectively), it can be concluded that the injection molding process allows the formation of an efficient percolation network in both directions.

### 3.2. Morphological Characterization

**Figure 3:**
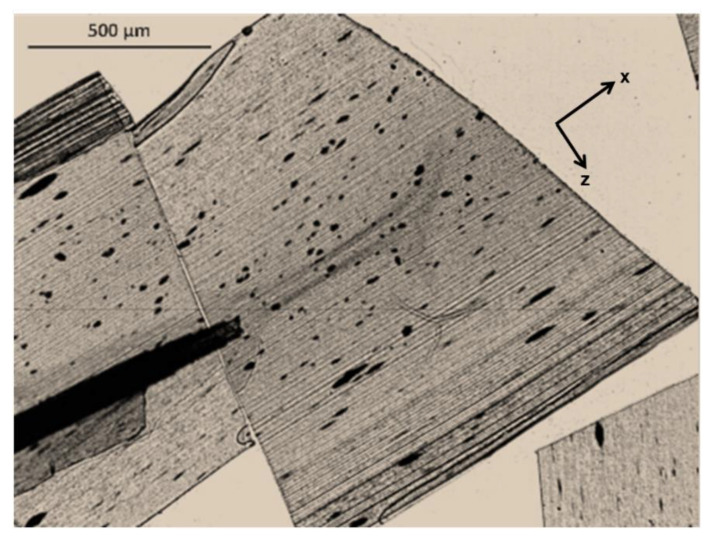
Optical micrograph of 3%-MWCNT nanocomposite, obtained from the entire thickness of the *xz* plane of the sample.

A morphological characterization was performed to experimentally investigate the obtained percolation network. As an example, Figure 3 shows the optical micrographs of the 3%-MWCNT nanocomposite obtained from the entire thickness of the *xz* plane of the sample. This content is particularly interesting since it is in the range of the percolation threshold in both the directions. A non-homogeneous morphology of the MWCNT clusters all through the thickness of the samples is clearly visible. In the core region, MWCNT clusters are typically smaller and have a more uniform shape, whereas in the skin layer they appear bigger, and more elongated along the in-plane direction.

**Figure 4:**
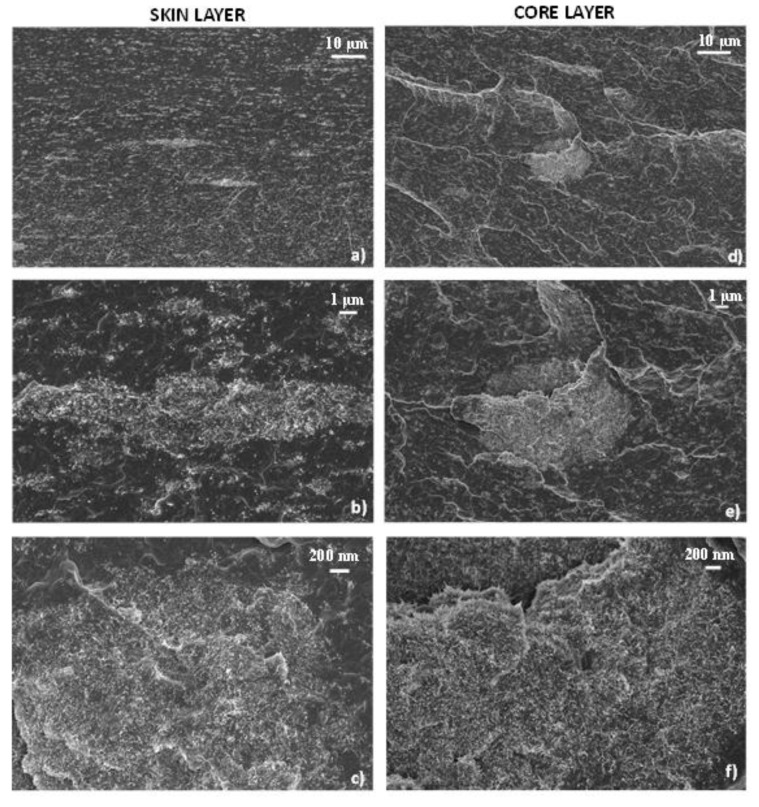
FESEM images of the cross-section of 3%-MWCNT nanocomposite. The micrographs were obtained at increasing magnifications from the skin layers (**a**–**c**) and from the core regions (**d**–**f**) respectively.

FESEM micrographs of the cross-sectional area of the 3%-MWCNT nanocomposite are reported in Figure 4. In these images, moving from the skin layer to the core region, the variation of morphological microstructure is evident and further confirms what has been observed in the optical microscopy image previously reported. Elongated and narrow MWCNT clusters are observable in the skin region (Figure 4a–c), while rounded and circular ones are visible in the core region (Figure 4d–f). Clustering formations are present with dimension ranging from 5–20 µm, even if a population of submicron clusters is also visible in both regions. As can be clearly seen, the bigger micrometric clusters as well as all the submicron clustering formations are aligned in the skin layer. At the same time, in the core region all the clusters–even the smaller ones–have a homogeneous circular shape. The inner morphology of these structures, both in the skin and in the core layers, shows MWCNT entanglements randomly oriented, with no preferential orientation of the single MWCNTs, regardless of the shape of the cluster itself. Moreover, the clusters seem to be impregnated by the polymer matrix, which is able to reach their internal areas. 

### 3.3. Effect of Processing Conditions

As a consequence of the morphological analysis and the electrical measurements, the electrical conduction mechanism is mainly regulated by the electron transfer via cluster interconnection. It is widely reported in literature that the conduction mechanisms of disordered systems are governed by two physical processes, such as classical hopping and quantum mechanical tunneling of charge carried over the potential barrier, separating two energetically favorable centers in a random distribution [41]. The tunneling is considered the primary conductive mechanism for polymer filled with conductive fillers such as MWCNTs. This behavior is described with a fluctuation-induced tunneling model [42,43]. This difference in the morphology of the specimens, due to the presence of a skin-core structure, is frequently observed on injection-molded samples [44,45] and it can be explained by the shear stress gradient in the through-thickness direction, which is produced by the flow of the molten polymer during the processing phase. With respect to the electrical behavior, as reported in a previous study by the same authors [21], the observed morphology of the core region typically leads to a more efficient conductive network than the one of the skin layers. This observation explains the lower percolation threshold and higher conductive plateau obtained in the X direction. Indeed, the through-thickness structure “skin layer/core region/skin layer”, which can be used to model the sample morphology, acts as a resistor made by the series of the three layers, when the electrical test is performed in the Z direction, and by the parallel of the same layers, when the electrical test is performed in the X direction. The use of Ohm’s law to this system perfectly justifies the obtained experimental results. The variation of the injection molding processing conditions typically modifies the shear stress of the molten nanocomposite and the related morphology. 

#### 3.3.1. Electrical Properties

**Figure 5:**
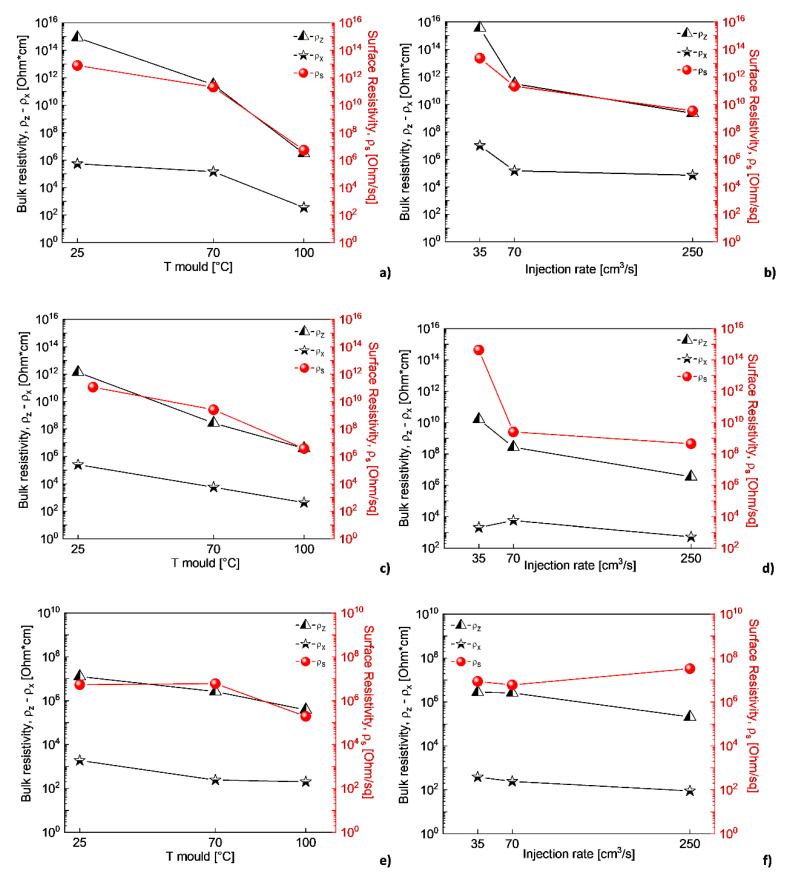
Effect of the mold temperature and injection rate on the electrical bulk and surface resistivity of: 2%-MWCNT nanocomposites, injection rate 70 cm^3^/s, and (**a**) mold temperature 70 °C (**b**); 3%-MWCNT nanocomposites, injection rate 70 cm^3^/s (**c**) and mold temperature 70 °C (**d**); 4%-MWCNT nanocomposites, injection rate 70 cm^3^/s (**e**) and mold temperature 70 °C (**f**).

Figure 5 shows the effect of the mold temperature and injection rate on both the bulk resistivity (ρ_z_ and ρ_x_) and the surface resistivity (ρ_s_), of the 2%, 3% and 4% MWCNT-based formulations. As it can be observed in Figure 5a,c,e, the increase in mold temperature leads to a reduction of bulk and surface electrical resistivity. This is particularly evident when the temperature is set at 100 °C and for the 2% and 3% MWCNT-based formulations, which are closer to the percolation threshold. 

The increase of the injection rate leads to a reduction of several orders of magnitude of the bulk and surface electrical resistivity of the nanocomposites (Figure 5b,d,f), moving from a fully insulating behavior to a partially conductive one. Nonetheless, based on the typical shear thinning behavior of polymers in their molten state, an increase of the flow rate can be most likely related to a reduction of the viscosity, with a resulting effect on morphology similar to the one produced by the increase of the mold temperature. 

#### 3.3.2. Rheology

**Figure 6:**
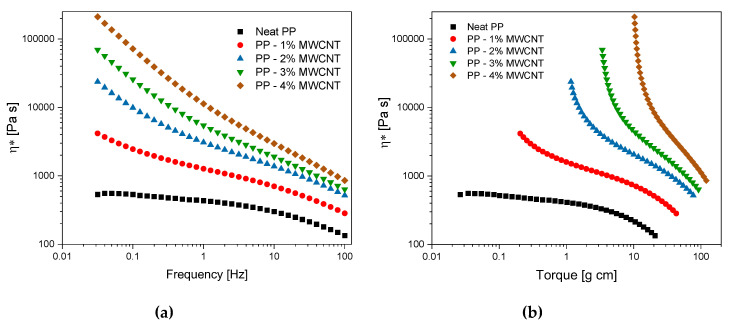
Viscosity of MWCNT nanocomposites as a function of (**a**) frequency and (**b**) torque.

**Figure 7:**
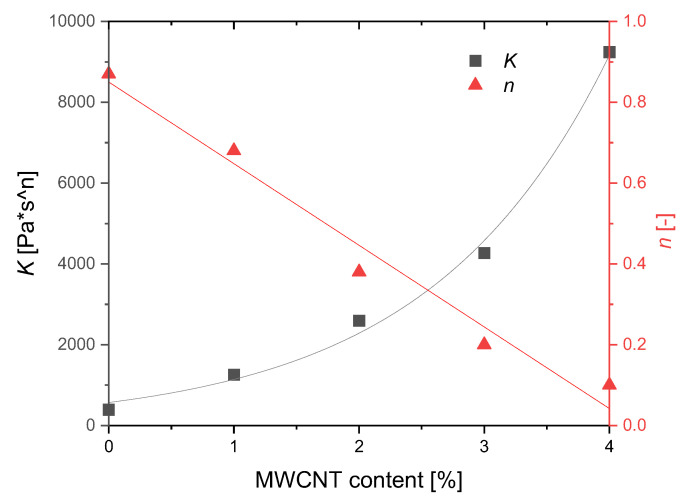
Flow consistency K and power-law *n* indexes as calculated by the fitting of the viscosity curves reported in Figure 6a.

A rheological characterization was performed, as explained in the experimental section. The main results are reported in Figure 6. The first graph (Figure 6a) shows the complex viscosity η* as a function of the oscillation frequency. As it can be observed, the viscosity of the nanocomposites is increasingly higher with increasing the MWCNT content. This increase is more pronounced in the low frequency region and it becomes less significant at higher frequencies. The tendency of the nanocomposites to change their rheological behavior from a liquid-like to a solid-like material is evident and it can be due to the formation of a filler network, increasingly denser with the MWCNT content [46,47]. Furthermore, all MWCNT-based samples show a more pronounced shear-thinning behavior if compared with the neat polymer. Indeed, particle-particle interactions are reduced due to the higher shear rate, and the effect of MWCNTs on the rheological behavior of the polymer is less evident in this frequency region (10–100 rad/s). On the contrary, the neat polymer exhibits a lower frequency dependency and a more pronounced Newtonian-like behavior, in accordance with the literature [48,49]. The formation of a filler network is also confirmed by the results reported in Figure 6b, which shows the viscosity as a function of the measured torque during the tests. These curves demonstrate that a yield stress occurs for the nanocomposites with 2 wt%, 3 wt% and 4 wt% MWCNTs. This feature is typical of complex fluids, in which the molten material does not flow unless a specific value of the applied stress. The tendency to a yield stress at the beginning of the flow can be explained with the overcoming of a physical percolation threshold [50,51]. The curves of the complex viscosity as a function of the oscillation frequency reported in Figure 6a have been fitted with the typical power-law equation η = Kω^(*n*−1)^, where K is the flow consistency index and n the power-law index. This equation is inspired by the Ostwald–de Waele relationship τ = K${\overset{\cdot }{\gamma }}^{n}$ that connects the shear stress to the shear rate of a fluid. Figure 7 reports the values of both K and n as a function of the MWCNT content. As it is possible to observe, a clear tendency of both the parameters can be observed, which have been highlighted with the over-position of fitting curves. The flow consistency parameter (K) increases exponentially with the MWCNT content, further confirming the effect of increasing viscosity with the presence of MWCNTs. 

The power-law index linearly decreases with MWCNT content. A power-law index lower than 1 indicates a shear thinning behavior of the fluid, while a power law index equal to 1 obtains the typical equation of Newtonian fluids, with no relation between shear rate and viscosity. In this case, a linear reduction of the index with increasing the MWCNT content clearly indicates that the shear thinning behavior is increasingly more pronounced with the filler fraction.

#### 3.3.3. Morphology

**Figure 8:**
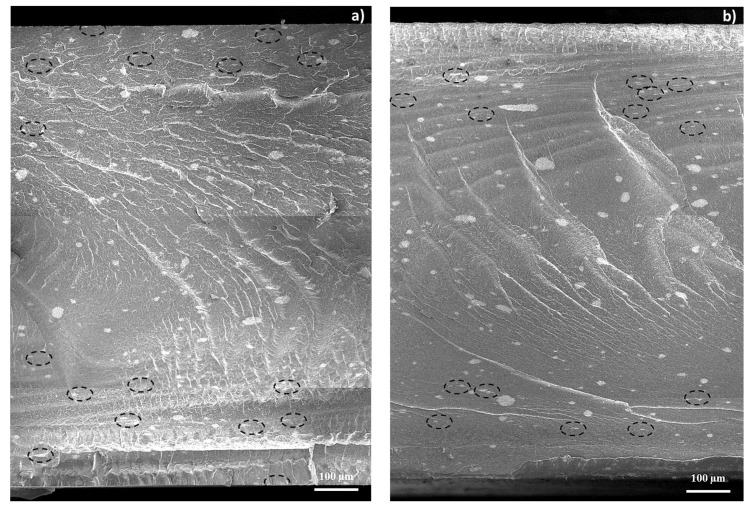
FESEM images of the cross-sectional area of 3%-MWCNT nanocomposites, manufactured at mold temperature 25 °C (**a**) and 100 °C (**b**), respectively (dashed black circles have been depicted to highlight some of the smaller clusters).

Figure 8 shows the cross-sectional area of the 3%-MWCNT nanocomposites produced at both 25 °C and 100 °C mold temperature. As it is possible to observe, FESEM images further confirm what previously observed regarding the skin-core morphology of the injection-molded samples (Figure 4). 

**Figure 9:**
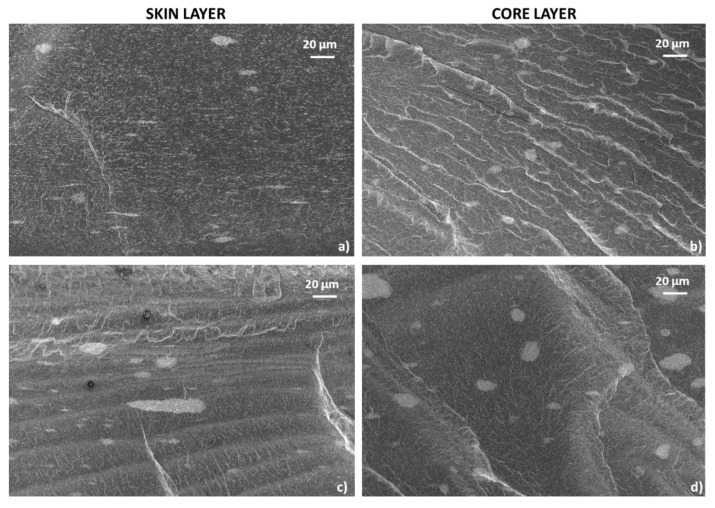
Enlargement of the FESEM images of the cross-section of 3%-MWCNT nanocomposite, as reported in Figure 8. The micrographs were obtained from the skin layers of the nanocomposites manufactured at mold temperature 25 °C (**a**) and 100 °C and (**c**) from the core regions of the nanocomposites manufactured at mold temperature 25 °C and (**b**) 100 °C (**d**), respectively.

Indeed, as observable in Figure 9, which shows larger magnification images of the cross-sectional area of the specimens, a majority of rounded clusters in the core region are visible, and they gradually become more elongated in the in-plane direction, passing in the skin layers. However, the observable morphological differences, in the samples manufactured at both mold temperatures, seem not to fully justify the strong effect on the electrical behavior.

#### 3.3.4. X-ray Diffraction (XRD)

**Figure 10:**
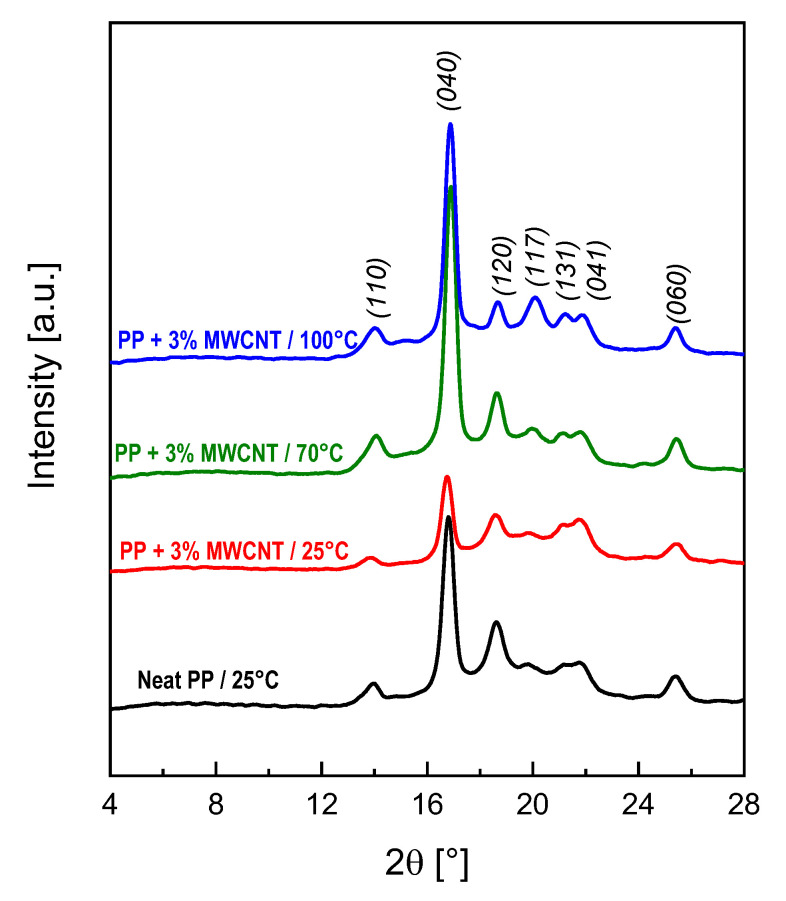
X-ray diffraction patterns of neat PP in comparison with the 3%-MWCNT nanocomposites manufactured at the three different mold temperature.

In this contest, XRD analysis was performed in order to shed light on the role of the different mold temperatures on the structure of the produced samples, with the ultimate goal of correlating a different crystalline structure with a potentially different electrical behavior. Although, it is well-known [35] that typically fillers tend to stay in the amorphous phase, the morphology of this phase is driven by the crystalline structure formed during the solidification phase. Figure 10 shows X-ray diffraction patterns of neat PP in comparison with the 3%-MWCNT nanocomposite at the three different mold temperatures. The XRD peaks at 2θ = 14.0°, 16.9°, 18.7°, 21.2°, 21.8° and 25.4° correspond to the *(110), (040), (120), (131), (041),* and *(060)* crystalline planes of α-phase, respectively [52]. Indeed, according to the literature, PP can crystallize according to three polymorphic crystallographic forms: monoclinic α-phase, hexagonal β-phase and triclinic γ-phase [33,53]. Each of α, β, and γ crystalline forms has its own distinctive peaks in the XRD patterns. The peak at 2θ = 20.1°, which corresponds to the *(117)* crystalline plane, is associated to the γ-phase. The XRD patterns, reported in Figure 9, show that the intensity of this peak increases with increasing the mold temperature. The amount of γ-phase (X_γ_) can be calculated by the Equation (3),

(3) $$X_{\gamma }=\frac{h_{\gamma }}{\left(h_{\gamma }+h_{\alpha }\right)}$$

where *h_γ_* and *h_α_* are the peak height of *(117)* and *(120)* planes respectively (the last one being selected as a reference for *α*-phase) [33,54]. The experimental value of *X_γ_* for the neat PP is 11%, while the values for the 3%-MWCNT nanocomposites, processed at different mold temperature, are reported in Table 1.

The data reported in Table 1 further confirm that the presence of γ-phase is increasingly higher with increasing mold temperature, indicating that the formation of this specific crystalline structure is favored by a slower cooling rate, which is obtained at higher mold temperature. This is in agreement with Mezghani et al. [55] that observed a similar behavior for the formation of the PP γ-phase when the crystallization is performed at a slower cooling rate.

This result, together with the electrical behavior reported in Figure 5, represents a clear indication that higher mold temperature (in particular equal to 100 °C) induces the formation of a crystalline structure, which leads to a rearrangement of a more efficient MWCNT network in terms of electrical conductivity performance. 

#### 3.3.5. Differential Scanning Calorimetry (DSC)

**Figure 11:**
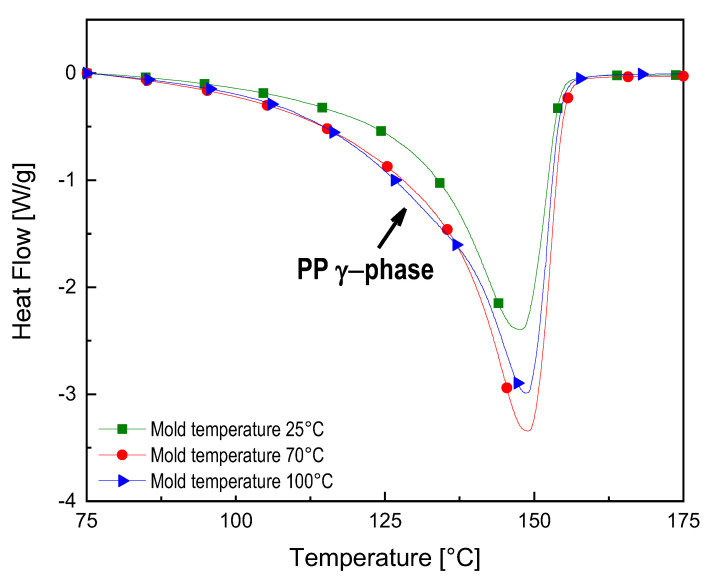
DSC thermogram of 3%-MWCNT nanocomposites manufactured at T25–70–100 °C (exo up).

Figure 11 reports the DSC curves of 3%-MWCNT nanocomposites, manufactured at 25–70–100 °C mold temperature. As it can be observed from the thermogram, a broad melting peak, constituted by a second peak in the temperature region between 120 °C and 145 °C, partially hidden by the main peak (at about 150 °C), is visible for the nanocomposites manufactured at mold temperature 70 °C and 100 °C. Zhu et al. [33] attributed this peak to the melting of the PP γ-crystals. Therefore, the growing broadness of this peak with the increase of mold temperature is consistent with the results obtained for the XRD measurements, where at the same processing condition an increase of X_γ_ in the 3 wt%-MWCNT occurs.

The evaluation of the degree of crystallinity of the neat PP manufactured at mold temperature 25 °C in comparison with the 3%-MWCNT nanocomposites, manufactured at mold temperature 25–70–100 °C has been performed. The X_c_ value of the neat PP is 35.4%, while the values for the 3%-MWCNT nanocomposites, processed at different mold temperature, are reported in Table 2. The increase of mold temperature up to 100 °C seems to slightly favor a higher degree of crystallinity. On the other hand, the samples manufactured at 25 and 70 °C as a mold temperature, allow a crystalline fraction of about 35%, with a difference between the two results which is fully in the range of the standard deviation experimentally evaluated.

As already shown in FESEM images (Figure 8 and Figure 9), there are no significant differences in the morphology of the main clusters. This means that the highly conductive network, related to the presence of a crystalline arrangement of γ-phase with the α-phase, is most likely associated with a more efficient inter-cluster connection induced by the shape of the amorphous phase, resulting from the melt crystallization, rather than significant modification of the intra-cluster morphology.

## 4. Conclusions

MWCNTs were successfully mixed with PP by melt-mixing in a co-rotating twin-screw extruder, and then used to manufacture samples by injection molding. The temperature of the mold and the injection rate were tuned in order to obtain samples with the same MWCNT content, but with potentially different properties. The electrical characterization has shown that an increase in the mold temperature and of the injection rate leads to a reduction of electrical resistivity. The correlation of the electrical properties with the morphology of these samples has been thoroughly studied. Optical and field emission scanning electron (FESEM) microscopies were used to observe the morphology of the MWCNT clusters. X-ray diffraction has shown that the increase of the mold temperature, which relates to a slower cooling rate, leads to the formation of the PP γ-phase. The decrease of the electrical resistivity can be associated to the presence of a larger fraction of a crystalline *γ*-phase, which seems to favor a more efficient inter-connection of MWCNT clusters, rather than significant modification of the intra-cluster morphology.

In conclusion, adjusting the injection molding processing conditions leads to a modification of the crystalline structure together with the inter-cluster connections, which results in tunable electrical properties. This phenomenon is particularly remarkable (variation of several orders of magnitude) when the MWCNT content is in the range of the percolation threshold.

The obtained results increase the awareness of the importance of the advanced process design of electrically conductive injection-molded components. The in-depth understanding of the possibility in tuning the electrical conductivity, and how it can be controlled by XRD, can provide an important step forward for the definition of optimized processing procedures.

## Figures and Tables

**Table 1:** Fraction of PP γ-phase in 3%-MWCNT nanocomposites.

Mold Temperature	X_γ_ (%)
25 °C	14
70 °C	18
100 °C	54

**Table 2:** Degree of crystallinity of 3%-MWCNT nanocomposites as a function of mold temperature.

Mold Temperature	X_γ_
25 °C	35.2 ± 1.3
70 °C	34.8 ± 0.9
100 °C	39.8 ± 1.1
